# Plasma membrane phospholipid phosphatase-related proteins as pleiotropic regulators of neuron growth and excitability

**DOI:** 10.3389/fnmol.2022.984655

**Published:** 2022-09-15

**Authors:** Joachim Fuchs, Shannon Bareesel, Cristina Kroon, Alexandra Polyzou, Britta J. Eickholt, George Leondaritis

**Affiliations:** ^1^Institute of Molecular Biology and Biochemistry, Charité – Universitätsmedizin Berlin, Freie Universität Berlin, Humboldt-Universität zu Berlin, and Berlin Institute of Health, Berlin, Germany; ^2^Department of Pharmacology, Faculty of Medicine, School of Health Sciences, University of Ioannina, Ioannina, Greece; ^3^Institute of Biosciences, University Research Center Ioannina, University of Ioannina, Ioannina, Greece

**Keywords:** phospholipid phosphatases, LPA, plasticity-related genes, filopodia, synaptic transmission, axonal regeneration, axonal development

## Abstract

Neuronal plasma membrane proteins are essential for integrating cell extrinsic and cell intrinsic signals to orchestrate neuronal differentiation, growth and plasticity in the developing and adult nervous system. Here, we shed light on the family of plasma membrane proteins phospholipid phosphatase-related proteins (PLPPRs) (alternative name, PRGs; plasticity-related genes) that fine-tune neuronal growth and synaptic transmission in the central nervous system. Several studies uncovered essential functions of PLPPRs in filopodia formation, axon guidance and branching during nervous system development and regeneration, as well as in the control of dendritic spine number and excitability. Loss of PLPPR expression in knockout mice increases susceptibility to seizures, and results in defects in sensory information processing, development of psychiatric disorders, stress-related behaviors and abnormal social interaction. However, the exact function of PLPPRs in the context of neurological diseases is largely unclear. Although initially described as active lysophosphatidic acid (LPA) ecto-phosphatases that regulate the levels of this extracellular bioactive lipid, PLPPRs lack catalytic activity against LPA. Nevertheless, they emerge as atypical LPA modulators, by regulating LPA mediated signaling processes. In this review, we summarize the effects of this protein family on cellular morphology, generation and maintenance of cellular protrusions as well as highlight their known neuronal functions and phenotypes of KO mice. We discuss the molecular mechanisms of PLPPRs including the deployment of phospholipids, actin-cytoskeleton and small GTPase signaling pathways, with a focus on identifying gaps in our knowledge to stimulate interest in this understudied protein family.

## Introduction

First identified as plasticity-related ecto-enzymes involved in antagonizing phospholipid-induced growth cone collapse (Bräuer et al., 2003), PLPPRs have emerged as a pleiotropic family of proteins. They regulate multiple independent processes during neuron growth and excitability and act through several independent cellular mechanisms. PLPPRs are integral membrane proteins with five identified family members (PLPPR1-5). They belong to the lipid phosphatase/phosphotransferase (LPT) family that modulate bioactive lipid phosphates including lysophosphatidic acid and sphingosine-1-phosphate (Sigal et al., 2005; Tang et al., 2015). It is of note that little is known concerning extracellular binding of lipids and/or activation, or details on how PLPPRs transfer signals to control cellular responses. What makes this family of proteins interesting is their association with cellular growth responses in a highly spatially and temporally organized manner. In this review, we aim to summarize the current knowledge of how PLPPRs regulate the specific, yet divergent, cellular processes that have been related to these membrane proteins.

### Structure and topology of the proteins

**FIGURE 1:**
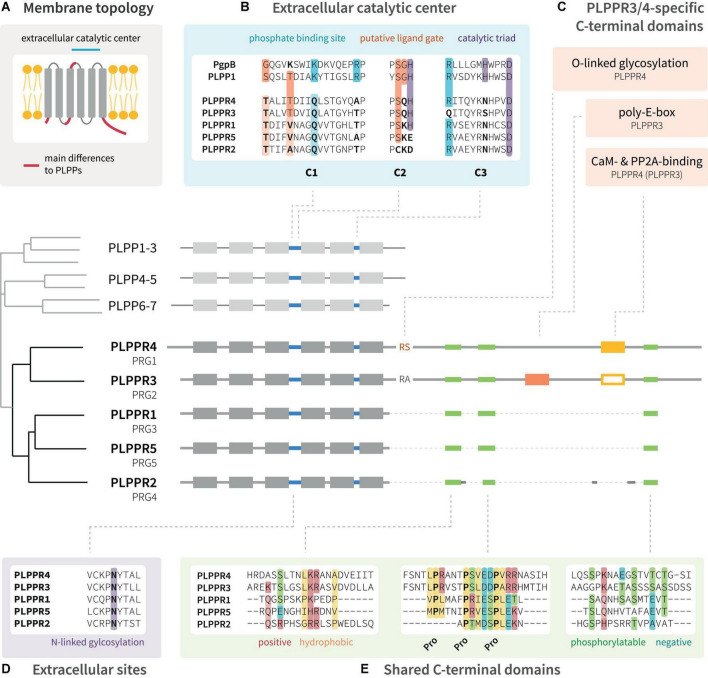
Domain structure of PLPPR family proteins. **(A)** PLPPRs are transmembrane proteins with six membrane-spanning domains; both the N-terminus and C-terminus are localized in the cytosol. **(B)** The extracellular catalytic center of PLPPRs shares some homology with PLPPs, but differs in critical catalytic and phosphate-binding residues. **(C)** PLPPRs differ from PLPPs in their C-terminus and cluster into a group of long-tail PLPPRs (PLPPR3, PLPPR4) and a group of short-tail PLPPRs (PLPPR1, PLPPR2, PLPPR5). PLPPR4 contains a cytosolic *O*-linked glycosylation site, and calmodulin and PP2A binding sites. The calmodulin and PP2A binding site is also found in PLPPR3 but has not been validated. The PLPPR3 sequence encompasses an unusual stretch of 20 glutamic acids. **(D,E)** All PLPPRs share an extracellular *N*-linked glycosylation site, a short sequence of positive and hydrophobic aa, a charged proline motif and a cluster of phosphorylation sites. Alignments as well as the cladogram were generated with UniProt sequences of mouse PLPPRs or PLPPs using the MAFFT algorithm available at EMBL-EBI (https://www.ebi.ac.uk/Tools/msa/mafft/).

Phospholipid phosphatase-related proteins are derived evolutionarily from the protein family of phospholipid phosphatases (PLPPs, Figure 1) (Sigal et al., 2005). They appear to emerge late in evolution from PLPPs with homologs first detected in nematodes (*C. elegans*^1^). PLPPs act as hydrolases, which catalyze the cleavage of phosphate from a bioactive lipid substrate (Zhang et al., 2000). PLPPs and PLPPRs share a conserved folding topology with six membrane spanning domains displaying the N- and C-terminal regions in the intracellular space (Figure 1A; Brindley and Waggoner, 1998; Sigal et al., 2005). While the extracellular loops in PLPPs contain the conserved catalytic domains C1, C2 and C3 (Waggoner et al., 1999), PLPPRs bear amino acid (aa) substitutions in several critical catalytic residues (Figure 1B; Sigal et al., 2005). PLPPRs evolved into a distinct set of proteins differing among each other by the length of their unstructured intracellular C-terminal domain (ICD). PLPPR1, PLPPR2 and PLPPR5 have an ∼50 aa ICD, while PLPPR3 and PLPPR4 present with a considerably longer ∼400 aa ICD (Figure 1; Bräuer and Nitsch, 2008). Although they differ in length, some regions, most notably a regularly spaced proline motif, are conserved in all ICDs. Intriguingly, PLPPR2 has extensive mutations in these shared C-terminal regions (Figure 1E). The long ICDs of PLPPR3 and PLPPR4 contain domains for binding to Calmodulin (CaM) and PP2A, and PLPPR4 possesses additionally an (intracellular) *O*-linked glycosylation site. A rarely occurring stretch of 20 consecutive glutamic acid residues within the ICD, the ‘poly-E-box,’ characterizes PLPPR3 (Figure 1C).

There has been a long-standing complication as the PRG nomenclature is frequently used to refer to PLPPRs, even though PRG officially denotes the proteoglycan family (Figure 1). The parallel usage of PLPPRs and PRGs has led to a conflicting numbering with PLPPR1 being PRG3, PLPPR2 being PRG4, PLPPR3 being PRG2, PLPPR4 being PRG1. Only PLPPR5 shares the same numbering with its PRG5 name. Additional complication comes from metazoan model organisms (*C. elegans*, *D. melanogaster*), where the PRG1/2 nomenclature corresponds to Piwi-related Argonaute proteins. Confusion has even spread to some antibody vendors, where irrelevant antibodies are associated with PRG1 or PRG2. All of the above combined have led to wrong citations of PLPPR functions (e.g., correction by Sun et al., 2022b) and disease-relevant papers connecting proteoglycan- or PLPPR-loss to unlikely molecular mechanisms. Standardizing the nomenclature will certainly minimize ambiguities and increase the visibility of this interesting protein family. In this review, we use the PLPPR nomenclature throughout.

## Expression and localization of phospholipid phosphatase- related proteins

Phospholipid phosphatase-related proteins are brain-enriched proteins with tightly regulated expression levels throughout different phases of development (Bräuer and Nitsch, 2008; Yu et al., 2015). Their restricted expression suggests roles at defined times in developmental programs of neurons. Furthermore, there is evidence that PLPPRs localize to distinct compartments of cells, indicating a spatial segregation of PLPPR functions. Although research on PLPPRs has to date mainly focused on glutamatergic hippocampal and cortical neurons, their expression and localization patterns suggest roles in other brain regions and cell types. In the following paragraphs, we describe the expression and localization patterns of PLPPRs *in situ*, in primary neuron cultures, and in the cell lines commonly used in the PLPPR literature.

### Expression in the central nervous system

**FIGURE 2:**
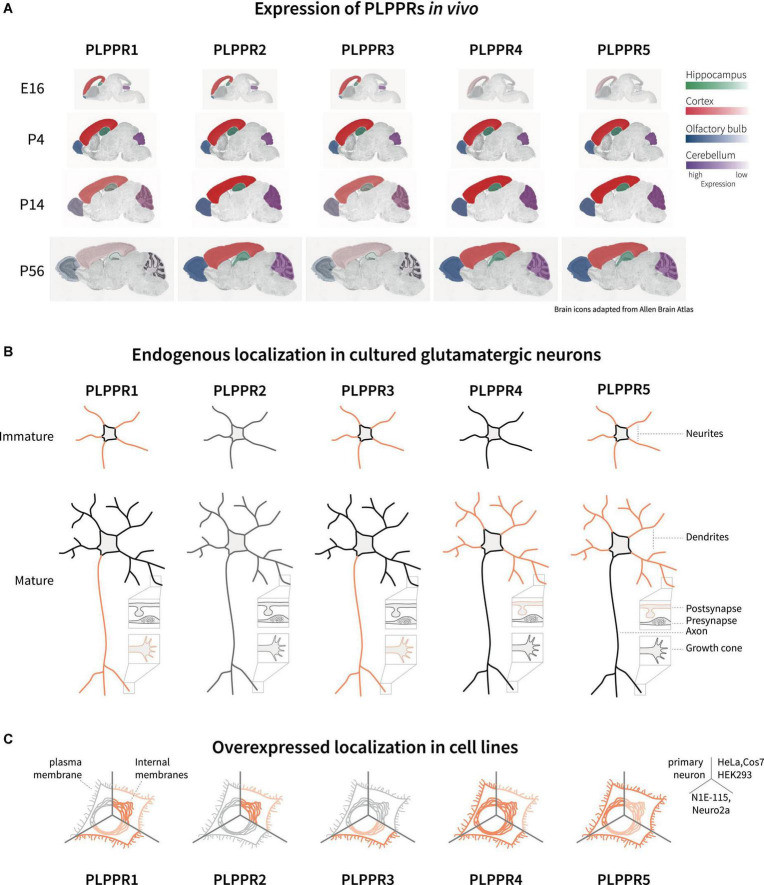
Expression and localization of PLPPRs *in vivo* and *in vitro*. **(A)** Expression of PLPPRs at different developmental and adult stages in the rodent brain. Color intensity correlates with relative expression levels in the hippocampus (green), cortex (red), olfactory bulb (blue), and the cerebellum (purple). **(B)** Localization of PLPPRs in different compartments (neurite, dendrite, pre-/post-synapse, growth cone) in cultured hippocampal/cortical neurons. **(C)** Subcellular localization of tagged-PLPPRs in plasma- and intracellular membrane compartments following overexpression in different cells. For details and references, please refer to text.

Analyses of the developing mouse and rat brains have shown that during early developmental stages at embryonic (E) days E14-E16, PLPPR1 mRNA is detected in the subventricular zone, the ventricular zone and the cortical plate, as well as the hippocampal anlage (Savaskan et al., 2004; Wang and Molnár, 2005; Figure 2A). Similarly, PLPPR5 expression is detectable at embryonic stages in the hippocampus (Coiro et al., 2014), with strong expression in the dentate gyrus (Broggini et al., 2010; Gross et al., 2022). *In situ* hybridization and immunoblotting analyses identified that PLPPR3 is expressed from E14.5 onward in thalamic and cortical areas (Cheng et al., 2016) with increasing levels of mRNA and protein expression from E16 until early postnatal stages (Figure 2A; Brosig et al., 2019). Expression analyses in rat cortex, hippocampus and cerebellum confirmed dynamic expression of PLPPR3, with high levels found at young postnatal stages (P7-P15) and reduced expression from around P21 onward (Brosig et al., 2019; Figure 2A).

In addition to embryonic expression, some PLPPRs are also detected after birth. Expression of PLPPR4 begins at E19 and persists during early adulthood at P30 (Bräuer et al., 2003; Liu et al., 2016; Figure 2A). Analyses of PLPPR4 expression using *in situ* hybridization and immunoblot shows high levels in hippocampus, cortex, cerebellum and subcortical striatal structures, in brainstem and spinal cord (Trimbuch et al., 2009; Tokumitsu et al., 2010; Yaguchi et al., 2012; Vogt et al., 2016). Prominent expression in adult animals is also seen for PLPPR5 in hippocampus, cerebellum and striatum while low PLPPR5 levels are detected in thalamus, hypothalamus, and pons (Gross et al., 2022). This overlap of PLPPR4 and PLPPR5 expression might indicate shared regulation, and indeed PLPPR4 and PLPPR5 genes are located within 300 kb distance in the mouse genome. PLPPR1 is detected at postnatal stages in layers II-VI throughout cortical regions (Wang and Molnár, 2005) and in the hippocampus, with a peak in mRNA expression around birth and high protein levels at P5-P10 (Velmans et al., 2013). The least well-studied member of the PLPPR protein family, PLPPR2, is expressed during E14 to P30 in hippocampus, neocortex, olfactory bulbs and cerebellum (Figure 2A). Compared to the dynamic expression of PLPPR1, PLPPR3, PLPPR4 and PLPPR5, the expression of PLPPR2 remains constant throughout development and does not change in the adult (Gross et al., 2021).

In addition to cortical and subcortical brain regions, PLPPR expression has been detected in the spinal cord. Antibody labeling identified the presence of PLPPR1 in murine spinal cord motor neurons of the ventral horn (Broggini et al., 2016). *In situ* hybridization of PLPPR5 mRNA in mouse spinal cords show high mRNA levels at P4 compared to lower expression in adult mice (Broggini et al., 2010).

Examination of the GABAergic interneuron markers GAD67, parvalbumin, calbindin and calretinin revealed no co-expression with PLPPR4 in the CA1 region of hippocampus. Furthermore, PLPPR4 did not colocalize with gephyrin, suggesting the overall absence of PLPPR4 from GABAergic synapses, at least in the adult hippocampus (Trimbuch et al., 2009). In contrast, PLPPR5 is likely to be expressed in both excitatory and parvalbumin-positive GABAergic neurons (Gross et al., 2022). Furthermore, several recent single cell RNAseq studies indicate expression of PLPPR1, PLPPR3 and PLPPR5 in adult murine cortical GABAergic interneurons (Zeisel et al., 2018; La Manno et al., 2021; Yao et al., 2021). Such expression in GABAergic neurons with a potentially different developmental expression time course compared to glutamatergic neurons is especially interesting given that studies on functions and expression of PLPPRs have so far focused on glutamatergic hippocampal and cortical neurons.

### Expression in other tissues and cells

In the brain, low expression of PLPPRs has been detected in astrocytes with additional expression of PLPPR1, PLPPR2, PLPPR3 and PLPPR5 in microglia (Velmans et al., 2013; Coiro et al., 2014; Gross et al., 2021). Similarly, mRNA of all PLPPR members is detected in immature and mature primary oligodendrocytes, with the exception of PLPPR2, which decreases in expression from immature to mature oligodendrocytes (Gross et al., 2022). In addition, qRT-PCR of PLPPR1 mRNA showed expression in mouse and rat astrocytes, as well as in rodent glioma cells, in contrast to microglial cells (Fan et al., 2014; Gross et al., 2021). *In vivo*, PLPPR4 and PLPPR5 proteins are not detected in GFAP-positive astrocytes (Trimbuch et al., 2009; Gross et al., 2022), but PLPPR5 seems to label a subtype of oligodendrocytes in the corpus callosum (Gross et al., 2022).

Besides brain, there have been reports of PLPPR expression in other tissues. For example, at transcript level, PLPPR3 is expressed in testis, ovary and lymph nodes (Sigal et al., 2005). A recent study showed prominent expression of PLPPR3 protein in spermatogonial stem cells, where it may serve as a surface marker that can be used for isolation of these cells (Tan et al., 2020). Similarly, mRNA expression of PLPPR1, PLPPR4 and PLPPR5 has been reported in the testis (Bräuer et al., 2003; Broggini et al., 2010). PLPPR5 mRNA is additionally detected in heart and lung (Coiro et al., 2014), while PLPPR1 transcripts are expressed in liver and kidney (Savaskan et al., 2004). Western blot analysis has confirmed PLPPR4 protein expression in testis using rat tissue homogenates (Tokumitsu et al., 2010). However, the expression of PLPPR1 protein in other mouse tissues was not confirmed (Velmans et al., 2013).

In addition, several transcriptome studies and experimental data indicate expression of PLPPRs in cancer cell lines and tumors. PLPPR1 has been shown to be downregulated in breast cancer (Bao et al., 2019) and in glioblastoma tissue (Aaberg-Jessen et al., 2018); in the latter, both up- and downregulated PLPPR1 levels are associated with shorter survival of glioblastoma patients (Fan et al., 2016). PLPPR5 appears also to be downregulated in a subtype of higher-grade glioblastoma (Stange et al., 2022). Interestingly, overexpression of PLPPR5 leads to a more benign tumor phenotype with decelerated growth and dysfunctional vascular architecture. In another transcriptome study, PLPPR1, PLPPR3 and PLPPR5 are among the top 25 most differentially expressed membrane proteins in pediatric cancer types (Orentas et al., 2012). PLPPR2 has been associated with several cancers including colorectal, pancreatic and breast cancer cell lines and tissues (Sagiv et al., 2008; Li et al., 2015; Talukder et al., 2016; Boonsongserm et al., 2019). For example, PLPPR2 expression is deregulated in colorectal cancer patient samples and cells (Boonsongserm et al., 2019), while in breast cancer samples, PLPPR2 shows high frequency of an aa substitution at T278 (PLPPR2 T278S), potentially worsening breast cancer outcome (Li et al., 2015). PLPPR4 mRNA as well as protein levels are upregulated in some gastric cancer cell lines and its upregulation in gastric cancer is associated with metastasis and poor prognosis (Zang et al., 2020). The mechanism by which PLPPR4 affects tumorigenesis appears to involve cell adhesion. Recently, PLPPR4 was identified as a tumor microenvironment-based prognostic marker for gastric cancer (Wang et al., 2022). Overall, these data indicate aberrant expression of some PLPPRs in cancer lines and tissue suggesting a link to tumorigenesis.

### Localization and trafficking

An important question concerns the localization of PLPPRs in polarized neurons. Membrane proteins localized to axons or dendrites engage in different cellular functions, for example pre- and postsynaptic modulation of synapses. Immunofluorescence analysis of PLPPR3 in E16 brains has consistently shown co-expression with the axonal marker L1 in the corticocortical and thalamocortical tracts (Cheng et al., 2016; Brosig et al., 2019). Although initially proposed as an axonal protein (Bräuer et al., 2003), immunofluorescence, biochemical fractionation and electron microscopy studies have identified PLPPR4 in the postsynaptic density of hippocampal glutamatergic synapses, while it is absent from presynaptic terminals. Including findings from similar localization studies for other PLPPRs, a consensus emerges that PLPPR1 and PLPPR3 are primarily localized in axonal membranes, while PLPPR4 and PLPPR5 are localized primarily in dendritic membranes (Velmans et al., 2013; Coiro et al., 2014; Yu et al., 2015; Broggini et al., 2016; Brosig et al., 2019; Gross et al., 2022; Figure 2B).

Both the trafficking to the plasma membrane as well as the subcellular localization and presentation at the plasma membrane are critical to understand the cellular function of membrane proteins. To date, there have only been few studies assessing the localization of PLPPRs in neurons or neuronal cell lines. In most cell lines studied, PLPPRs are not readily detected at the protein level with existing antibodies (Sigal et al., 2005; Iweka et al., 2020; Gross et al., 2021; our unpublished data). As such, most studies have utilized transient overexpression or stable cell lines to study PLPPR localization to the different cellular membrane systems. In Cos-7, HeLa or N1E-115 and Neuro2a neuroblastoma cell lines, overexpressed PLPPR1, PLPPR3, PLPPR4 and PLPPR5 were located at variable levels to the plasma membrane (Bräuer et al., 2003; Savaskan et al., 2004; Sigal et al., 2007; van Coevorden-Hameete et al., 2015; Brosig et al., 2019; Tilve et al., 2020), while PLPPR2 overexpressed in HEK293 was observed mostly in intracellular compartments (Gross et al., 2021; Figure 2C). It is highly likely that the trafficking modality and spatial organization of PLPPRs at the plasma membrane imposes additional and complementary mechanisms for controlling cellular PLPPRs responses.

Interestingly, co-overexpression of different PLPPR family members increases expression of PLPPRs and enhances their plasma membrane localization (Yu et al., 2015; Brosig et al., 2019). For example, PLPPR1 is an efficient regulator of plasma membrane localization of PLPPR3 and PLPPR5 (Yu et al., 2015). This co-operation between PLPPR members depends on multimer formation resulting in homomeric or heteromeric PLPPRs complexes (Yu et al., 2015; Brosig et al., 2019). Strikingly, structured illumination microscopy (SIM) of PLPPR3 using specific antibodies in hippocampal neurons showed puncta at the axonal plasma membrane (Brosig et al., 2019). Furthermore, super resolution microscopy of co-overexpressed PLPPR1 and PLPPR5 or PLPPR3 showed both puncta formation at the plasma membrane in neuroblastoma cell lines (Yu et al., 2015; Brosig et al., 2019). Thus, homo- or heterodimerization may potentially be a driver in the trafficking and targeting of PLPPRs to the plasma membrane, although this has not been tested directly.

In conclusion, based on a variety of experimental approaches followed by different laboratories, a pattern emerges with two groups of PLPPRs sharing expression and localization patterns. On the one hand, PLPPR1 and PLPPR3 express strongest during early development, predominantly localize to the neuron’s axon (Figure 2B) and are downregulated in mature neurons (Figure 2A). On the other hand, both PLPPR4 and PLPPR5 increase in expression after birth, and predominantly localize to dendritic compartments (Figures 2A,B). Interestingly, PLPPR expression partners do not correspond in their overall domain architecture. PLPPR3 and PLPPR4 share most structural similarities, with both encompassing a long ICD. PLPPR1 and PLPPR5 (and to some extent PLPPR2) make up the second structural group characterized by presence of a short ICD (Figure 1). In conjunction with the ability of PLPPRs to form heteromeric complexes (Yu et al., 2015), this may argue for an ‘axon group’ and a ‘dendrite group’ that both utilize one member of the PLPPR family with short ICD and one PLPPR family member with a long ICD to exert their functions.

## Generation of membrane protrusions and filopodia

Filopodia are small actin rich plasma membrane protrusions that modulate cellular processes such as migration, adhesion, neurite outgrowth and growth cone guidance (Gallop, 2020; Wit and Hiesinger, 2022). During neuronal morphogenesis, they also serve as precursors for neurites (Dent et al., 2007; Gallo, 2013), axonal branches (Kalil and Dent, 2014), and dendritic spines (Ziv and Smith, 1996). Although it is difficult to morphologically distinguish the different types of membrane protrusions such as conventional filopodia, cytonemes and retraction fibers (Svitkina, 2018), there is general agreement in the field that PLPPRs induce filopodial-type membrane protrusions.

### Evidence of filopodia formation

**FIGURE 3:**
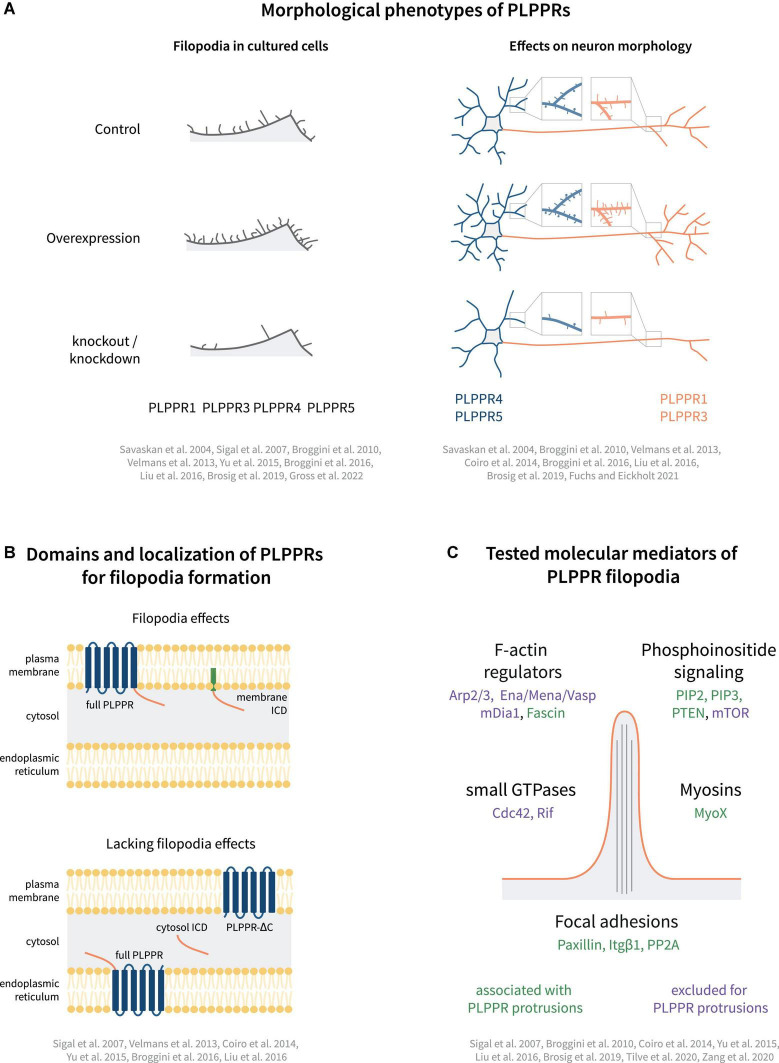
Models of PLPPR-based membrane protrusions. **(A)** Effect of PLPPRs overexpression/knockout on filopodia formation in cells, dendrite/dendritic spine morphology (blue) and axon morphology (orange). **(B)** Plasma membrane localization, is required for induction of filopodia. **(C)** Cellular regulators involved in PLPPR-induced membrane protrusions, filopodia formation and filopodia stabilization/adhesion. Note that regulators written in green letters are associated with PLPPR-based membrane protrusions, whilst factors written in purple have been tested in a specific experimental paradigm but can be excluded as mediators/regulators. For details, please refer to text.

PLPPR1, the most studied PLPPR family member, shows strong activity in inducing membrane protrusions. Following overexpression, PLPPR1 localizes uniformly to filopodial shafts and occasionally enriches in filopodial tips (Sigal et al., 2007). It induces filopodia in non-neuronal (Savaskan et al., 2004; Sigal et al., 2007; Velmans et al., 2013) and neuronal cell lines (Savaskan et al., 2004; Yu et al., 2015; Broggini et al., 2016), as well as in hippocampal neurons (Velmans et al., 2013). Furthermore, PLPPR1 overexpression has been shown to induce filopodia *in vivo* (Broggini et al., 2016). Knockdown of PLPPR1 leads to a reduction in the number of filopodia in cancer cell lines (Sigal et al., 2007), as well as in hippocampal neurons (Velmans et al., 2013). Taken together, these data strongly indicate that PLPPR1 is involved in the induction of filopodia (Figure 3A).

PLPPR5 is another member of the PLPPR family of proteins that induces filopodia. Overexpression of PLPPR5 promotes the formation of plasma membrane protrusions in P19 carcinoma and N1E-115 neuroblastoma cells (Broggini et al., 2010), as well as in cortical and hippocampal neurons (Broggini et al., 2010; Coiro et al., 2014). PLPPR5 overexpression in mature hippocampal neurons stimulates the formation of dendritic filopodia and spines independent of neuronal activity (Gross et al., 2022). However, in immature hippocampal neurons, PLPPR5 downregulation does not affect the number of filopodia (Coiro et al., 2014). Overall, and despite the high variability of cellular phenotypes in some studies, there is a consensus for efficient filopodia or membrane protrusion activity which can be linked to PLPPR1 and PLPPR5 (Figure 3A).

In the context of generating filopodia, the family members PLPPR3 and PLPPR4 are less studied, although data indicates similar functions (Figure 3A). For example, PLPPR4 expression increases filopodia density in HEK293 cells (Liu et al., 2016), while PLPPR3 expression in embryonic stem cell derived motor neurons induces the formation of filopodia in axons (Brosig et al., 2019). Accordingly, hippocampal neurons isolated from PLPPR3 KO mice develop fewer axon filopodia without changing filopodia length (Brosig et al., 2019; Fuchs et al., 2020; Fuchs and Eickholt, 2021). The only PLPPR family member currently not linked to filopodia or other membrane protrusions is PLPPR2 (Gross et al., 2021). However, given that overexpression did not result in plasma membrane targeting, it is difficult to assess if PLPPR2 can promote filopodia formation.

Few studies have analyzed PLPPR-induced changes in filopodial length, an important aspect to be considered for distinguishing between induction of filopodia versus regulating their elongation. It was reported that PLPPR1 and PLPPR5 induce longer filopodia in non-neuronal cells when compared to GFP-transfected control cells (Savaskan et al., 2004; Broggini et al., 2010). However, analysis of gain-of-function and loss-of-function filopodia phenotypes in neurons suggests that PLPPR3 specifically affects filopodia numbers but not filopodia length (Brosig et al., 2019).

### Molecular mechanisms of filopodia formation

Cells utilize multiple signaling cascades to generate filopodia, which include signaling mediators such as the small GTPases Cdc42 and Rif (Nobes and Hall, 1995; Pellegrin and Mellor, 2005), actin regulators such as ENA/VASP, Arp2/3 and formins (for a review see Yang and Svitkina, 2011), and proteins interacting with membrane lipids, for example IRSp53 (reviewed in Ahmed et al., 2010). For a recent review, focusing specifically on the mechanisms of filopodia formation, we would like to refer to excellent reviews by Jacquemet et al. (2015) and Gallop (2020). In the following sections, we discuss the different mechanisms that have been implicated in filopodia generated by PLPPRs.

#### Structure-function relationships

Plasma membrane localization of PLPPRs appears to be a prerequisite for filopodia formation. Several groups investigated the involvement of the PLPP catalytic domain by generating non-conservative substitutions of residues in the C2 and C3 motifs (S198T, H200G, R246E; Figure 1B), which trapped PLPPR1 in intracellular membranes and abolished filopodia formation (Sigal et al., 2007). Similarly, mutations in these residues dispersed PLPPR5 from the plasma membrane and affected normal PLPPR5 induced membrane protrusions (Coiro et al., 2014). However, introducing an E195H mutation in PLPPR5 (as present in PLPPR1, PLPPR3, PLPPR4, and PLPPs), restored prominent plasma membrane expression in neurons, and induced membrane protrusions in both soma and neurites. An *N*-glycosylation deficient variant of PLPPR1 (Figure 1D) did not localize to the plasma membrane and failed to induce filopodia (Velmans et al., 2013). Together these results provide a strong argument that plasma membrane localization of PLPPRs is essential for filopodia formation (Figure 3B).

Furthermore, a body of experimental data suggests the importance of the intracellular domain (ICD) for PLPPR-induced filopodia formation (Figure 3B). A truncated PLPPR1 variant lacking the ICD (PLPPR1 aa 1-279) localizes to the plasma membrane and intracellular compartments in a manner similar to full length PLPPR1, but is incapable of inducing filopodia (Sigal et al., 2007; Broggini et al., 2016). However, another study suggested that a similar truncated PLPPR1 (aa 1-282) retains the ability to induce membrane protrusions (Yu et al., 2015). Interestingly, the isolated cytosolic PLPPR1 ICD (PLPPR1 aa 280/283-325) cannot induce filopodia (Yu et al., 2015; Broggini et al., 2016), but mimics full length PLPPR1 filopodia induction when inserted into the plasma membrane via a myristoylation tag (Broggini et al., 2016). The truncated PLPPR5 and PLPPR4, similarly, are unable to induce membrane protrusions (Coiro et al., 2014; Liu et al., 2016). In addition, the cytosolic PLPPR4 ICD (PLPPR4 aa 338-766) alone is insufficient in inducing filopodia. The above data suggest that the PLPPR-transmembrane domains as well as membrane localized ICDs are required for induction of filopodia (Figure 3B).

#### Role of small GTPases

The Rho GTPases Cdc42 and Rif regulate distinct pathways of filopodia formation (Passey et al., 2004; Goh and Ahmed, 2012). PLPPR-induced filopodia formation appears to be independent of Cdc42 (Sigal et al., 2007; Broggini et al., 2010). When compared to the filopodia induced by constitutively active Cdc42, PLPPR1-induced filopodia are longer, thinner and more persistent (Sigal et al., 2007). In addition to this, co-expression of PLPPR1 or PLPPR5 with dominant-negative Cdc42 does not impair filopodia formation (Sigal et al., 2007; Broggini et al., 2010). Furthermore, there are no changes in the ability of PLPPR1 to induce filopodia when Cdc42 function is inhibited (Sigal et al., 2007). These results indicate that PLPPRs induce filopodia independently of Cdc42. This also applies to downstream Cdc42 signaling (Sigal et al., 2007; Broggini et al., 2010), since a number of actin modulators like Arp2/3 (Gautreau et al., 2022), Mena/VASP (Breitsprecher et al., 2008; Faix and Rottner, 2022) and formins (Courtemanche, 2018; Le et al., 2020) appear to be dispensable for PLPPR1- and PLPPR5-dependent filopodia formation (Figure 3C).

In contrast to Cdc42, little is known concerning Rif-dependent filopodia formation, which utilizes both formin homology proteins mDia1 and mDia2 (Goh et al., 2011). Filopodia induced by Rif are similar to filopodia induced by PLPPR1 in terms of length and thickness (Sigal et al., 2007). However, co-expression of PLPPR1 with dominant-negative Rif does not impair PLPPR1-induced filopodia formation, demonstrating that PLPPR1 does not operate upstream of Rif. Since PLPPR5 was shown to act independent of mDia1, which is also an effector in Rif-dependent pathway, it is possible that PLPPR1 and other family members act independent of mDia1 to form filopodia (Figure 3C).

#### Role of phosphoinositide signaling

The phosphoinositides PI(4,5)P2 and PI(3,4,5)P3 are essential upstream regulators of the F-actin reorganization machinery that drive formation of filopodia and lamellipodia in cells, including neurons (Balla, 2013). Furthermore, PI(3,4,5)P3 is the second messenger of the PI3K-AKT-mTOR signaling pathway, known to regulate physiological and pathological growth, cellular metabolism and proliferation in cells (reviewed in Porta et al., 2014).

PLPPR3 induces axonal filopodia in a PI(3,4,5)P3-dependent manner, as treatment with PI3K inhibitor LY294002 abolished the ability of PLPPR3 to induce filopodia in embryonic stem cell motor neurons (Brosig et al., 2019). PLPPR3 likely exerts its effect on PI(3,4,5)P3 signaling by regulating the phosphatase PTEN, which directly antagonizes PI3K signaling by hydrolyzing PI(3,4,5)P3. PLPPR3 was shown to interact directly with PTEN, to localize PTEN to the plasma membrane and to attenuate PTEN’s phosphatase activity. Morphological analysis showed that PTEN knockdown reversed the branching deficit in PLPPR3 KO neurons. Taken together, these results suggest that PTEN and PI(3,4,5)P3 are essential for PLPPR3-induced axonal filopodia and branches (Brosig et al., 2019; Fuchs et al., 2020). PTEN has also been shown to interact with PLPPR1 (Yu et al., 2015). In the same study, mass spectrometry and co-immunoprecipitation experiments revealed an interaction also with PTEN’s downstream effector mTOR. Interestingly, co-expression of PLPPRs increased the phosphorylation of S6 ribosomal protein, a downstream target of mTOR. However, blocking the mTOR pathway with rapamycin had no effect on PLPPR-induced protrusions. Another member of the PLPPR family has also been proposed to interact with phosphoinositide signaling pathways. It has been suggested that full-length PLPPR5, likely via its ICD, binds phosphoinositides *in vitro* (Coiro et al., 2014; Figure 3C). Although the nature of this interaction is uncertain and its role in PLPPR5 filopodia inducing activity unknown, direct experiments with PLPPR3 in microscale thermophoresis binding assays have suggested binding to both PI(3,4,5)P3 and PI(4,5)P2 (our own unpublished data). These preliminary findings may suggest additional direct interactions of PLPPRs with membrane phosphoinositides. It is plausible that such interactions, besides providing a link to phosphoinositide-signaling pathways (Brosig et al., 2019) or clustering phosphoinositides into plasma membrane microdomains (McLaughlin and Murray, 2005), may also serve a regulatory role in PLPPR stability or function, potentially analogous to the known regulatory roles of phosphoinositides in facilitating ion channel function (Balla, 2013).

#### Role of cell adhesion

A further cellular mechanism that may be exploited by PLPPRs during the generation (or stabilization) of filopodia is cell adhesion (Fischer et al., 2019). Indeed, activation of integrin, a major component of focal adhesions, promotes filopodia formation in cancer cells (Jacquemet et al., 2016). Nascent focal adhesions are sites of myosin X-dependent filopodia formation and elongation in Cos-7 cells (He et al., 2017). A recent review (Gallop, 2020) proposed that once filopodia are formed, the subsequent formation of adhesive structures in filopodia shaft and tip may promote filopodia length and lifetime.

In this respect, it is interesting, that both PLPPR1 and PLPPR4 overexpression increase adhesion to fibronectin-coated surfaces, and PLPPR4 overexpression increases additionally adhesion to laminin-coated surfaces (Liu et al., 2016; Tilve et al., 2020; Figure 3C). Furthermore, overexpression of PLPPR4 leads to increased filopodia density and surface expression of activated β1-integrin (ITGB1) in HEK293 cells, while PLPPR4 KO antagonizes ITGB1 activity and reduces dendritic spine density in hippocampal neurons (Liu et al., 2016). However, PLPPR4 does not interact directly with ITGB1. Instead, it was shown that the cytoplasmic calmodulin-binding domain of PLPPR4 interacts with PP2A to establish the adhesome that activates ITGB1. In agreement with this model, activation of PP2A using fingolimod (FTY720) rescues spine density, demonstrating that PLPPR4-based adhesion complexes control spine density. However, it is worth mentioning that FTY720 has a considerable number of other targets in the cell (White et al., 2016). PLPPR4 involvement in adhesion has also been implicated in cancer metastasis. Cell migration relies on filopodia, which sense the surrounding environment. Increased filopodia density and migration, both of which depend on adhesion to the extracellular matrix (Jacquemet et al., 2015), are hallmarks of cancer metastasis (Arjonen et al., 2011). PLPPR4 knockdown leads to decreased migration, invasion, wound healing and adhesion in gastric cancer cell lines, while overexpression has the opposite effect (Zang et al., 2020). PLPPR4 appears to impinge on the expression of focal adhesion components including several α-integrins, p-FAK, p-Src, p-Akt and MMP2 via the transcription factor Sp1 (Zang et al., 2020). In the same vein, overexpression of Sp1 in PLPPR4 knockdown cells rescues the cell migration, invasion, and adhesion phenotypes. PLPPR4 knockdown also inhibited tumor progression *in vivo*. Taken together, these results indicate that PLPPR4 promotes gastric cancer metastasis via Sp1-integrin α signaling.

In addition to cellular adhesion, PLPPR1 overexpression decreases cell migration and causes cells to leave behind actin-less ‘trailing fibers.’ The molecular mechanisms were linked to decreases in Rac1 activity, and impaired maturation of nascent focal adhesion complexes that may account for the increased adhesion and decreased migration phenotype (Tilve et al., 2020). PLPPR1 may also associate with ITGB1, potentially promoting the establishment of focal adhesions (Yu et al., 2015). Although association of adhesions and filopodia is variable (Gallop, 2020), adhesion has clear mechanistic roles in stabilizing filopodia, and furthermore, recent work suggests that adhesion can also drive filopodia initiated branching morphogenesis (Fischer et al., 2019).

#### Molecular composition of phospholipid phosphatase-related protein-induced filopodia

Phospholipid phosphatase-related protein-based filopodia may be characterized by distinct molecular compositions. Although PLPPRs appear to generate filopodia in a Cdc42-independent manner, they are – similar to most cellular filopodia – based on actin filaments and devoid of microtubules (Yu et al., 2015). Filopodia induced by Cdc42 have been shown to contain focal adhesion proteins such as paxillin (Nobes and Hall, 1995) or VASP (Krugmann et al., 2001; Sigal et al., 2007) and the actin-based molecular motor protein myosin X (Tokuo and Ikebe, 2004; Sousa and Cheney, 2005), which are typically located at the tip of the protrusions. In contrast, the PLPPR1-labeled filopodia lack paxillin and VASP at their tips; instead, these proteins were found at the base of these filopodia (Sigal et al., 2007). However, myosin X is also found at the tips of PLPPR1-labeled filopodia (Sigal et al., 2007; Yu et al., 2015). The actin bundling protein fascin is considered as marker of filopodia and many cells require fascin-bundled F-actin to generate filopodia (Vignjevic et al., 2003). Fascin was found in the shafts of PLPPR1-induced filopodia, raising the intriguing possibility that PLPPRs generate a distinct filopodia type in cells. However, the molecular composition of filopodia may differ depending on the cell type, and, to date, such analyses have not been undertaken in primary neurons.

In summary, PLPPRs induce changes in cell morphology that are predominantly associated with filopodia formation. How this family of proteins governs these changes remains an open question, since none of the well-described pathways appears to influence PLPPR-dependent filopodia formation. Alternatively, instead of distinct signaling pathways and proteins governing protrusion dynamics, filopodia may form in response to diverse upstream signaling involving stochastic combinations of actin regulators (Dobramysl et al., 2021). This idea seems to be in agreement with current data that has failed to pinpoint the involvement of any individual well-known filopodia-inducing proteins in the formation of PLPPR-based filopodia.

## Phospholipid phosphatase-related proteins and lysophosphatidic acid signaling

Owing to the homology with PLPPs there has been considerable interest in the possible involvement of PLPPRs in neuronal LPA signaling. LPA plays essential roles in cortical development, myelination, pain, synapse transmission and plasticity, and axonal growth (Yung et al., 2015; Birgbauer, 2021).

Lysophosphatidic acid acts via numerous receptors suggesting a significant redundancy in signaling. Besides a group of six characterized GPCRs termed LPAR1-6, LPA also interacts with purinergic receptors, nuclear transcription factors and TRP-like channels (Nieto-Posadas et al., 2012; Jang et al., 2014; Yung et al., 2015). LPA-induced LPAR1/6 signaling involves G_*s*_, G_*i/o*_, G_*q/*11_ and G_12/13_ proteins and controls cellular proliferation via RAS/ERK1/2, cell survival via PI3K/AKT, cell migration via PI3K/RAC, changes in cell shape via RhoA/ROCK and calcium mobilization via G_*q*_/PLC pathways (Kumagai et al., 1993; Kranenburg et al., 1999; Fang et al., 2000; Mills and Moolenaar, 2003). The main source of extracellular LPA is autotaxin (ATX), a secreted Phospholipase D ectoenzyme, which hydrolyzes extracellular lysophospholipids, mainly lysophosphatidylcholine, to LPA (Tokumura et al., 1999; Herr et al., 2020). However, LPA is also produced independently via a phospholipase A1/A2 pathway acting on phosphatidic acid (Sonoda et al., 2002; Aoki et al., 2008). LPA is present at low nanomolar to micromolar concentrations in the CNS and cerebrospinal fluid (CSF) (Yung et al., 2014) and is rapidly cleared from fluids, with a half-life of 2–3 min in blood (Salous et al., 2013). Dephosphorylation of LPA leads to *in situ* inactivation, which is executed by members of the PLPP family (Tang et al., 2015).

**FIGURE 4:**
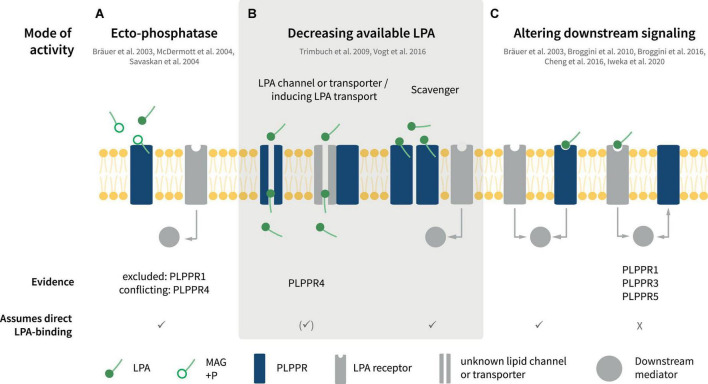
Putative modes of PLPPR interaction with LPA signaling. **(A)** PLPPRs may inactivate LPA via ectophosphatase activity. **(B)** PLPPRs may limit extracellular LPA in a direct or indirect manner, functioning as transporters or scavengers or by influencing an unknown LPA transporter. **(C)** PLPPRs impinge on LPA intracellular signaling either directly or indirectly, by inducing or inhibiting LPAR-mediated downstream signaling. Note that several, but not all of the modes presented imply direct binding of LPA to PLPPRs. For details, please refer to text.

Interestingly, LPA exerts both protrusive and retractive effects in neuronal shape and morphology (Sheng et al., 2015; Yung et al., 2015). This is largely because of its potency in inducing changes on actin filament dynamics and microtubule organization depending on the specific LPAR repertoire of neuron cell types. Neuronal LPA responses controlling migration, neurite retraction, cell rounding, growth cone collapse as well as axonal branching are attributed, at least in part, to one or more of LPAR1-6. For example, classical LPA-induced growth cone retraction involves LPAR1/2 receptors (Yung et al., 2015). Similarly, LPAR3 contributes partially to LPA-induced branching in neuronal cell lines and rodent neurons (Furuta et al., 2012). However, analysis of individual or even triple KO animals have not produced convincing data for LPAR1/2/3 being essential for axonal retraction or branching *in vitro* or *in vivo* (Birgbauer and Chun, 2010; Birgbauer, 2015, 2021; Yung et al., 2015). Instead, atypical LPA receptors like the TRP-like channel TPRM2 has been suggested to be responsible for some LPA responses, such as neurite retraction (Jang et al., 2014). Interestingly, at least some of the functions mediated by PLPPRs involve LPA signaling. Considering this contribution of PLPPRs in transducing or controlling LPA effects in neuron physiology, there have been many reports suggesting different modes of function for individual PLPPRs (Figures 4A–C).

### Role as lysophosphatidic acid phosphatases

Upon identification of PLPPRs and in light of their homology with PLPPs, PLPPRs were considered to possess LPA phosphatase activity. Despite initial reports suggesting LPA-ectophosphatase activity in PLPPR4 by measuring the product of LPA dephosphorylation, monoacylglycerol (MAG) in cell supernatants and membranes (Bräuer et al., 2003; Figure 4A), ectophosphatase activity was not detectable in subsequent experiments for PLPPR4 (McDermott et al., 2004) or PLPPR1 (McDermott et al., 2004; Savaskan et al., 2004). Considering that no additional data have been published for other PLPPRs, the agreement is that PLPPRs do not exhibit LPA phosphatase activity (Strauss and Bräuer, 2013). This notion is consistent with the changed catalytic motifs C1-C2-C3 in PLPPRs. Thorough inspection of PLPP and PLPPR alignments suggests that the PSGH-loop, which serves as a ligand gate (Tong et al., 2016), would be likely blocked, while the predicted phosphate binding residues are severely disturbed in PLPPRs. Nevertheless, the putative lipid-tail binding and pore-forming residues may be preserved (Figure 1B). Thus, it seems plausible that at least some PLPPRs may have retained direct binding to LPA or similar lipid-like molecules.

### Regulation of lysophosphatidic acid signaling pathways

**FIGURE 5:**
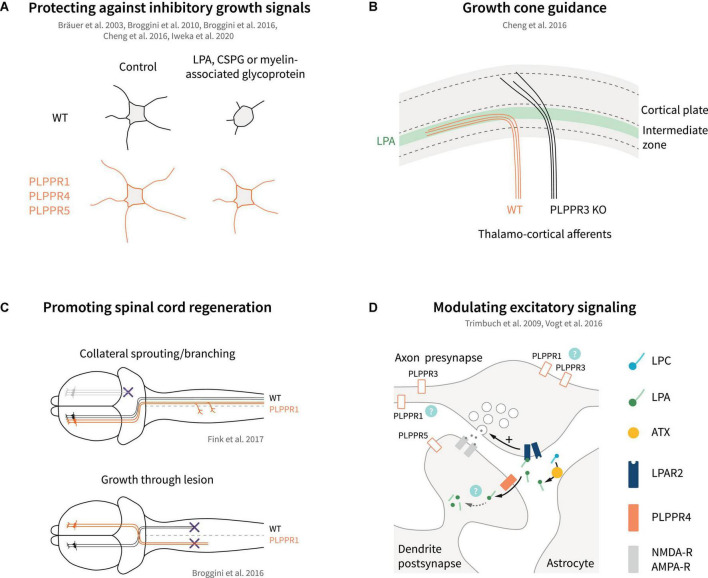
Models of PLPPR modulation of neuronal growth, guidance, regeneration and excitatory signaling. **(A)** Overexpression of several PLPPRs overcomes neuronal growth inhibition mediated by established inhibitory signals (LPA, CSPGs, myelin-associated glycoprotein). **(B)** PLPPR3 alters LPA-sensitivity of thalamocortical neurons to enter the developing cortex, thereby affecting axon guidance. **(C)** PLPPR1 overexpression improves re-innervation of neuron in the corticospinal tract in spinal cord injury models by increasing branching in uninjured neurons and by promoting extension of axons through the lesion. **(D)** PLPPR4 affects neurotransmitter release at excitatory synapses by reducing available LPA to act on presynaptic LPA-receptors. Possible roles of other PLPPRs are highlighted by question marks. For details, please refer to text.

Numerous studies have suggested that PLPPR1, PLPPR4, and PLPPR5 – following overexpression in neuronal cell lines or neurons – antagonize LPA downstream signaling and its effects on cellular morphology and neurite retraction (Bräuer et al., 2003; Broggini et al., 2010, 2016; Iweka et al., 2020; Figure 4C, see also Figure 5A). LPA causes neurite retraction and axon collapse mostly via the Rho/ROCK pathway (van der Bend et al., 1992; Tigyi et al., 1996; Kranenburg et al., 1999). PLPPR1 has been shown to reduce LPA- and serum dependent RhoA activation and to diminish LPA-induced ROCK-dependent phosphorylation of myosin light chains, myosin phosphatase 1 (MYPT1), as well as the ERM proteins Ezrin, Radixin and Moesin (Iweka et al., 2020). The proposed mechanism involves engagement of RhoA in inactive complexes with RhoGDI upon overexpression of PLPPR1. Although the details and specificity of interactions have not been delineated, these experiments suggest that exogenous PLPPR1 impedes LPA-induced dissociation of RhoA from RhoGDI (Iweka et al., 2020). In a similar manner, overexpression of PLPPR5 reduces LPA-activated RhoA in cell lines, particularly at low submicromolar LPA concentrations (Broggini et al., 2010). Thus, a pattern emerges that PLPPRs may function as a fine-tuning device for LPA-induced RhoA activity. However, it is not known how PLPPR1 and PLPPR5 modify the strength of LPA-induced RhoA/ROCK signaling and how the relationship to parallel LPAR-engagement of the Rho/ROCK pathway is achieved. From published data, it would appear that an interaction with LPAR-RhoA/ROCK signaling represents a constitutive function of PLPPR1 and PLPPR5 although this has not been formally tested.

The changes imposed by PLPPR1 and PLPPR5 on LPA-induced RhoA/ROCK pathway activation have been instrumental in explaining the resistance of PLPPR1 or PLPPR5-overexpressing cells toward LPA-induced neurite retraction (Broggini et al., 2010, 2016; Iweka et al., 2020). A similar effect has been reported for PLPPR4-overexpressing neuroblastoma cells (Bräuer et al., 2003) but, in comparison to PLPPR1 and PLPPR5, PLPPR4 appears to function in a different mode (see below). PLPPR3, on the other hand, has been proposed to function in a directly opposite manner to PLPPR1 and PLPPR5. Specifically, extending axons of thalamocortical explants confronted with a polarized concentration of LPA cannot invade the LPA-rich zone. In contrast, PLPPR3 KO thalamocortical axons appear to be able to cross the LPA zone, suggesting that the repulsive effect of LPA is mediated by PLPPR3 (Cheng et al., 2016). LPA is a known chemorepellent that causes growth cone collapse (Birgbauer and Chun, 2010). PLPPR3 KO thalamocortical neurons exposed locally to low concentrations of LPA do not exhibit the typical turning response of WT neurons but seemed to be attracted by LPA instead. This appears to be an LPA-specific response since PLPPR3 KO axons were repelled by Sema3A (Cheng et al., 2016), another chemorepellent operating in this system. Interestingly, PLPPR3 KO growth cones are insensitive – even at high concentrations – to undergo the characteristic LPA-induced collapse seen in WT neurons (Cheng et al., 2016). These results suggest that PLPPR3 functions as an atypical LPA receptor or LPA sensor that enables axonal LPA responses (Figure 4C). A possible mechanism responsible for this effect may involve interaction of PLPPR3 with Radixin, an ERM family protein. Radixin is known to function as a membrane-cytoskeletal linker which binds to F-actin and regulates its dynamics (Fehon et al., 2010). PLPPR3 and Radixin interact via multiple sites in the C-terminal tail of PLPPR3 in close proximity with CaM-binding and PP2A binding homology regions of PLPPR4 (Figure 1C), and the interaction is increased upon LPA stimulation. Loss of PLPPR3 results in reduced Rho/ROCK-dependent phosphorylation of ERM proteins, at basal conditions and upon LPA treatment (Cheng et al., 2016). It is still uncertain whether PLPPR3 similarly functions to enable axonal responses to LPA in other types of neurons (i.e., cortical or hippocampal). Nonetheless, it is likely that this LPA-sensing role of PLPPR3 relates to its effects on axonal filopodia and branches under basal conditions, an exciting possibility that requires further studies.

### Role as lysophosphatidic acid transporters

The first characterized PLPPR, PLPPR4 exhibits a different mode of function toward LPA signaling. Pioneering studies from the Nitsch and Vogt groups demonstrated that PLPPR4 is necessary and sufficient for LPA cellular uptake, and they presented PLPPR4 as potential LPA transporter or scavenger (Figure 4B). The first indication of this function came when WT hippocampal neurons were incubated with a fluorescent analog of phosphatidic acid (NBD-PA) (Trimbuch et al., 2009). In this assay, PLPPR4 KO neurons appeared to exhibit a 50% reduction of NBD-PA uptake compared to WT neurons. These results were later verified with fluorescent LPA uptake assays in neurons and in cell lines stably expressing PLPPR4 (Vogt et al., 2016). Furthermore, mass spectrometry experiments showed increased C17-LPA uptake (an unnatural analog of LPA) and its major metabolite C17-MAG in stably expressing PLPPR4 cell lines compared to control cells. Interestingly, PLPPR4 heterozygous hippocampal neurons showed an intermediate activity in LPA uptake compared to WT, suggesting a gene-dose-dependent effect. Although the structural details of the PLPPR-mediated transport function are not yet known, Vogt et al. (2016) have characterized a PLPPR4 mutant that abolishes LPA uptake activity. Cells expressing PLPPR4 R346T, a mutation located in the C-terminus proximal to the 6th transmembrane region (Figure 1C), show diminished ability to take up LPA compared to wild-type PLPPR4 despite similar plasma membrane localization. Surprisingly, the deleterious effect of the R346T mutation appears to be due to reduced *O*-glycosylation of the neighboring S347 residue. It appears that phosphorylation does not play a role in this response since both phospho-mimetic and phospho-ablating S347 mutants are deficient in LPA uptake. Although it is still unknown how the R346T mutation affects *O*-glycosylation of S347 and how LPA is transported across the membrane, the fact that PLPPR4 induces LPA transport is well supported by data. Intriguingly, this RS motif is not conserved in the short ICD PLPPRs (Figure 1C) and PLPPR3 contains an A residue next to R, resembling the PLPPR4 S347A mutant that lacks LPA transporter activity (Vogt et al., 2016), suggesting that LPA transport is a PLPPR4 specific function.

## Phospholipid phosphatase-related proteins in developmental growth and regeneration

### Neurite outgrowth, axonal and dendritic branching

There is substantial evidence that PLPPRs govern neurite and/or branch formation (Figure 3A). These effects likely relate to filopodia-inducing activities of PLPPRs as filopodia are known precursors of neurites and axonal/dendritic branches and dendritic spines (Sekino et al., 2007; Mattila and Lappalainen, 2008; Gallo, 2013; Leondaritis and Eickholt, 2015; Sainath and Gallo, 2015). PLPPR1 overexpression in neuroblastoma cells increases the number and length of neurites (Broggini et al., 2016), while PLPPR3 expression in stem-cell derived motor neurons led to a PI3K-dependent formation of multiple axons per cell. However, PLPPR3 KO did not affect the number of neurites in hippocampal neurons (Brosig et al., 2019). PLPPR5 knockdown in immature rat cortical neurons decreases the number of neurites (Broggini et al., 2010). The involvement of RhoA-Rho kinase signaling pathway has been investigated for PLPPR5-induced neurite formation. Co-expression of constitutively active RhoA with PLPPR5 abolished the formation of neurites seen in PLPPR5 expressing cells, suggesting that PLPPR5 may act upstream of RhoA or independently of it.

There is conflicting evidence if PLPPRs also regulate neurite growth. It was reported that knockdown of PLPPR5, but not PLPPR1, in neuronal cells inhibits neurite extension (Broggini et al., 2010; Velmans et al., 2013). However, PLPPR1 overexpression in cortical neurons results in increased neurite lengths and axonal branches (Fink et al., 2017). PLPPR3, on the other hand, appears to exert a different control on axonal branching and growth. Expression of PLPPR3 increases axonal branches, while PLPPR3 loss results in fewer axonal branches (Brosig et al., 2019). Interestingly, the latter is accompanied by an increase of the primary axon length. A detailed analysis of branch generation patterns in WT vs. PLPPR3 KO neurons suggests that the deficiency in filopodia formation seen in immature hippocampal neurons directly translates into fewer axonal branching events from filopodia with an overall decrease of branch stability (Fuchs and Eickholt, 2021). As such, PLPPR3 is the only PLPPR where the induction of axonal branches later in development is directly and almost etiologically related to the induction of filopodia in earlier stages.

Phospholipid phosphatase-related protein family members with expression patterns that peak later in development (i.e., PLPPR4 and PLPPR5) are involved in controlling dendritic arborization and formation of dendritic spines (Figure 3A). Cell culture experiments show that PLPPR4 KO hippocampal neurons have significantly fewer high-order dendritic branches (Liu et al., 2016). Moreover, PLPPR4 KO neurons show decreased dendritic spine density compared to wild type neurons, and overexpression of PLPPR4 in WT neurons significantly increased spine density. Importantly, PLPPR4 does not affect overall spine morphology, as the length and area of spines are not altered in the PLPPR4 KO (Liu et al., 2016). Along the same line, overexpression of PLPPR5 in mature hippocampal neurons increases dendritic spine density while knock-down of endogenous PLPPR5 using shRNA reduces dendritic spine density (Coiro et al., 2014). It is likely that the neurite and branch outgrowth activity of PLPPRs involves LPA and/or other extracellular growth inhibition signals and associated pathways. This feature is discussed in more detail in subsequent sections.

### Axon guidance

Axon guidance is the process where neuronal axons navigate to their correct (intermediate or final) targets, guided by different classes of extracellular signals (Tessier-Lavigne and Goodman, 1996). Several guidance molecules have been discovered, such as Netrins that can act both as chemoattractant via DCC receptor and chemorepellent via UNC-5 receptor (Kennedy et al., 1994). Downstream of netrin, DCC interacts with TRIM9 (Winkle et al., 2014) and TRIM67 (Boyer et al., 2018), E3 ubiquitin ligases that localize to filopodia tips and function in axon projection (Menon et al., 2021). Interestingly, TRIM67 interacts with PLPPR4 and PLPPR3 following overexpression in HEK293 cells and was shown to stabilize PLPPR4 protein levels, enhancing neurite outgrowth responses in N1E-115 cells (Yaguchi et al., 2012). In addition to this, PLPPR4 interacts with TRIM67 along filopodia tips, in axons, and in growth cones of cortical neurons (Menon et al., 2021). It is hypothesized that PLPPR3 and PLPPR4 may regulate TRIM-dependent changes in PI(3,4,5)P3 membrane levels and netrin-dependent branches (Menon et al., 2021). Altogether, these recent studies strengthen the finding of a regulatory role of PLPPRs during axon guidance. Indeed, PLPPR3 is essential for correct guidance of thalamocortical axonal projections into the intermediate zone during development (Cheng et al., 2016; Figure 5B). Proper guidance in this case depends on autotaxin-derived LPA and protein-protein interactions between PLPPR3 and radixin. Accordingly, adult PLPPR3 KO mice exhibit dampened neuronal activity at the cortical projection site as well as impaired local information processing as manifested by whisker-dependent sensory discrimination (Cheng et al., 2016).

### Neuronal regeneration

Neuronal regeneration after trauma is a complex process and interventions aimed at engaging the intrinsic capability of neurons for survival and regenerative growth and/or modifying the signaling mechanisms that respond to inhibitory molecules are actively pursued (Fawcett and Verhaagen, 2018; Griffin and Bradke, 2020). Interestingly, there are several lines of evidence that PLPPRs may impinge on regeneration processes following CNS injury.

Regenerative sprouting is an endogenous neuronal mechanism that takes place following axonal trauma (Griffin and Bradke, 2020), or in response to overstimulation of neuronal networks, for example in epilepsy (Cavarsan et al., 2018). In the latter case, abnormal regenerative sprouting may be viewed as a pathological feature associated with aggravated seizure development (Cavarsan et al., 2018). PLPPR4 was originally identified by screening for genes upregulated in the hippocampus during regenerative axon sprouting after transection of entorhinal axons in adult rodents (Bräuer et al., 2003). Subsequent studies have suggested the fluctuation of expression levels of PLPPR1, PLPPR4 and PLPPR5 during regenerative sprouting in the hippocampus upon recovery from toxic insults such as kainic acid injection (Savaskan et al., 2004), or drug-induced developmental seizures (Ni et al., 2010, 2011, 2013). However, inconsistencies in the experimental protocols and lack of evidence that altered PLPPR expression contributes to regenerative sprouting precludes any definite answer in these studies. On the contrary, a functional association of PLPPR1 with regenerative sprouting has been uncovered in corticospinal motor neurons (Fink et al., 2017). PLPPR1 was identified as one of the genes that are significantly upregulated in intact sprouting neurons compared to quiescent intact neurons after pyramidotomy. In addition, overexpression of PLPPR1 significantly increased neurite growth and branching as well as regeneration of axons after crush *in vitro*. *In vivo*, pyramidal neuron-specific PLPPR1 overexpression increased sprouting of intact resident corticospinal axons after pyramidotomy (Fink et al., 2017; Figure 5C). Interestingly, a similar effect was also observed after systemic pharmacological inhibition of LPAR1, suggesting the functional coupling of PLPPR1 to LPA-LPAR1 signaling (Fink et al., 2017). LPA levels are significantly increased upon neuronal trauma (Santos-Nogueira et al., 2015), and both LPAR1 and LPAR2 have been shown to be responsible for LPA-induced microglia/macrophage activation and demyelination during secondary damage after spinal cord contusion injury (Santos-Nogueira et al., 2015; López-Serrano et al., 2019). These effects have been primarily ascribed to microglial LPA signaling (Santos-Nogueira et al., 2015; López-Serrano et al., 2019); instead, PLPPR1 appears to regulate LPA-LPAR1 signaling in neurons and reinforces plasticity of intact neurons after injury (Fink et al., 2017).

In addition to regenerative sprouting upon trauma, enhancement of neuron survival as well as counteracting chondroitin sulfate proteoglycan (CSPG)- and myelin-associated inhibition of axonal growth are essential during regeneration (Fawcett and Verhaagen, 2018; Griffin and Bradke, 2020). Neurotrophins and PI3K/PTEN-mediated mTOR signaling have been shown to boost neuron survival and axonal regrowth in several *in vivo* studies (Liu et al., 2010; Griffin and Bradke, 2020; Nieuwenhuis and Eva, 2022), while PTEN also directly contributes to proteoglycan and myelin-induced inhibition of growth cones (Henle et al., 2013). Considering the association of PLPPR3 with PTEN and PI3K signaling (Brosig et al., 2019; Fuchs et al., 2020), an influence on neuronal survival and axonal regrowth is likely, but this has not been tested directly. Similarly, PLPPR1 has emerged as a strong candidate for inducing regeneration via counteracting proteoglycan and myelin-associated inhibition (Broggini et al., 2016; Iweka et al., 2020; Figure 5A). Overexpression of PLPPR1 in hippocampal neurons counteracts the inhibitory effects of CSPGs and myelin on neurite outgrowth and a similar effect is observed in neuroblastoma cells and LPA-induced neurite retraction (Broggini et al., 2016; Iweka et al., 2020). Interestingly, PLPPR1 appears to counteract the effect of LPA on Rho GTPase activation and downstream signaling via interactions with RhoGDI (Iweka et al., 2020). It is also likely that counteraction of growth inhibition by PLPPR1 might represent an indirect effect, since PLPPR1 overexpression increases also the cellular adhesion on inhibitory substrates such as chondroitin sulfate proteoglycans (Tilve et al., 2020). However, it should be emphasized that these results were obtained from gain-of-function experiments with PLPPR1 (i.e., overexpression in cell lines) and there is currently no evidence that PLPPR1 counteracts Rho-mediated inhibition at the endogenous levels in primary neurons. Notwithstanding these uncertainties, the overexpression of PLPPR1 in Thy1.2 driven PLPPR1 transgenic mice enhances regenerative axonal sprouting of motor cortical neurons after spinal cord injury and results in partial functional recovery of motor behavior in Schnell-swim-tests (Broggini et al., 2016). The studies by Broggini et al. (2016), Fink et al. (2017) and Iweka et al. (2020) clearly establish PLPPR1 as a neuronal membrane protein that facilitates regeneration of axons after trauma (Figures 5A,C). Whether this applies to other PLPPRs, either alone or in complexes with PLPPR1, awaits further studies. Furthermore, the fact that in most studies so far PLPPR1 appears to be equally effective in counteracting LPA-, proteoglycan- and myelin-dependent inhibition of growth (Broggini et al., 2016; Iweka et al., 2020) suggests a more general mechanism of function than the LPA/LPAR association suggested for other PLPPRs (i.e., PLPPR4).

Although there have been no conclusive studies on PLPPR-dependent neuronal survival or apoptosis, it has been reported that knockdown of PLPPR4 in neural stem cells decreases neuron viability *in vitro*, supposedly via induction of apoptotic death (Hashimoto et al., 2013). It is noteworthy that the H253A mutant of PLPPR4, which disables the conserved LPA binding motif of PLPPRs and PLPPs (Figure 1B) and prevents PLPPRs from plasma membrane localization, cannot support PLPPR4-dependent neuronal survival (Hashimoto et al., 2013). Whether this function is unique for PLPPR4 and whether it depends on LPA and LPARs remains to be studied. However, recent studies have suggested aggravated phenotypes upon hypoxia/ischemia insults and drug-induced seizures in PLPPR5 KO mice, potentially due to PLPPR5-dependent neuron survival (Sun et al., 2021, 2022a; Wang et al., 2021).

## Phospholipid phosphatase-related proteins in synaptic transmission

The first evidence that PLPPRs can function as regulators of synaptic transmission came from studies analyzing PLPPR4 function. Electron microscopy and synaptosomal fractionation revealed localization at the postsynaptic density of hippocampal glutamatergic synapses, while PLPPR4 is absent from presynaptic terminals (Trimbuch et al., 2009). As a postsynaptic membrane protein, PLPPR4 regulates functions of LPA and its LPAR receptors in glutamatergic synapses. LPA has neuromodulatory roles in both glutamatergic and GABAergic neurons (Roza et al., 2019; Birgbauer, 2021). Early studies showed that LPA enhances NMDA-evoked currents in CA1 hippocampal neurons (Lu et al., 1999), while later studies suggested LPA-dependent short-term depression of both glutamatergic and GABAergic synapses in hypoglossal motor neurons (García-Morales et al., 2015). The LPA effects on synaptic transmission occur primarily via LPAR1 and LPAR2 receptors (Trimbuch et al., 2009; Choi and Chun, 2013; García-Morales et al., 2015; Roza et al., 2019). Interestingly, PLPPR4 KO mice suffer from juvenile epileptic seizures from around postnatal day 20 onward and exhibit a mortality rate of 50% at 3–4 weeks (Trimbuch et al., 2009; Unichenko et al., 2016; Vogt et al., 2016). The epileptic seizures correspond to increases in excitatory synaptic transmission, which is readily observed in CA1 pyramidal neurons, with no effects on inhibitory events (Trimbuch et al., 2009). Of note, hyperexcitability of CA1 pyramidal neurons upon PLPPR4 loss correlates with PLPPR4 gene dosage, with PLPPR4 heterozygous neurons exhibiting an intermediate phenotype (Trimbuch et al., 2009). Detailed analysis of the hyperexcitability phenotype revealed that PLPPR4 functions by dampening LPA-LPAR2 signaling in glutamatergic synapses (Figure 5D). LPA is synthesized by astrocyte-derived autotaxin in glutamatergic synaptic clefts (Thalman et al., 2018), and acts on presynaptic LPAR2 receptors by increasing glutamate release in CA1 hippocampal neurons (Trimbuch et al., 2009; Roza et al., 2019). Postsynaptic PLPPR4 appears to function as LPA transporter and/or scavenger that removes LPA from the synaptic cleft (Trimbuch et al., 2009; Vogt et al., 2016; Figure 5D).

PLPPR4 is not the only member of the PLPPR family associated with seizures. PLPPR5, which is positioned on the same chromosome region within 300 kb distance from the PLPPR4 gene in humans and rodents, has also been associated with epilepsy, albeit in a drug-induced animal model. In these experiments, pilocarpine, a muscarinic agonist was used to induce seizures in juvenile mice and seizure susceptibility was studied in the surviving adult mice after injection of penicillin, a beta-lactam antibiotic (Wang et al., 2021). Although the experimental approach differs markedly from the PLPPR4 studies described in the previous paragraph, PLPPR5 KO mice were found to be more prone to epilepsy with a lower seizure latency compared to WT mice (Wang et al., 2021). Interestingly, PLPPR5 has been also shown to localize to dendrites of mature (DIV14) hippocampal neurons (Coiro et al., 2014), and likely in spines *in vivo* although it is excluded from the PSD (Gross et al., 2022). Contrary to PLPPR4, however, electrophysiological analyses of PLPPR5-deficient hippocampal neurons *in vitro* suggest hypoactivity, with a decrease in mEPSCs frequency but normal mEPSCs amplitude (Coiro et al., 2014). This hypoactivity is likely mediated by the reduced numbers of excitatory synapses quantified by vGlut1/GluR2 overlapping puncta on dendrites (Coiro et al., 2014). Unfortunately, there are no mechanistic molecular details whether PLPPR5 (or other PLPPRs) functions in a manner analogous to PLPPR4 in synaptic transmission (Figure 5D).

Further analysis of PLPPR4 KO mice suggests association of PLPPR4 with human neurodevelopmental diseases. On the one hand, electrophysiological studies of WT and PLPPR4 KO mice show a developmental switch from hypo-excitability to hyper-excitability in the somatosensory barrel cortex during postnatal developmental stages (PND16-19 to PND25-31), suggesting a developmentally regulated role of PLPPR4 in synapse function (Unichenko et al., 2016). Intriguingly, PLPPR4 KO mice that survive to adolescence, grow to display deficits in whisker-mediated sensory perception (Unichenko et al., 2016). On the other hand, behavioral analysis of PLPPR4 heterozygous mice, which are viable and seizure-free, suggest stress-related behavioral changes and altered resilience against psychiatric disorders (Vogt et al., 2016). These phenotypes depend on the LPA transporter-like, postsynaptic function of PLPPR4, since decreases in pre-pulse inhibition of the startle response in PLPPR4 heterozygous mice is reversed after intraperitoneal administration of PF8380, a potent inhibitor of the LPA-synthesizing enzyme autotaxin (Vogt et al., 2016). Furthermore, the PLPPR4 R346T variant detected as a single nucleotide polymorphism in humans, and associated with an endophenotype for psychiatric disorders, is a loss of function mutant for the LPA transporting activity of PLPPR4 (Vogt et al., 2016). Regardless of whether other PLPPRs are involved in LPA uptake, PLPPR4 deficiency emerges as a novel correlate to endophenotypes for psychiatric disorders, schizophrenia and resilience to stress.

A somewhat similar connection is seen in PLPPR1 KO mice. Preliminary studies show that PLPPR1 KO female mice exhibit hypoactivity and general anxiety, heightened sensitivity to fear and impaired pre-pulse inhibition of the startle response (Iweka, 2018). These findings further highlight the potential contribution of PLPPR deficiency to neuropsychiatric diseases. Whether effects of PLPPR1 deficiency directly depend on synaptic LPA synthesis and/or LPA-LPAR2 signaling (as is the case for PLPPR4) is not known.

Dendritic spine dynamics have been repeatedly linked to synaptic plasticity and often serve as a functional output that underlies deficits in processes associated with learning and memory. Interestingly PLPPR4 KO neurons exhibit reduced spine density that is readily observed in the CA1 and DG regions in young (P12-19) and adult animals (Liu et al., 2016). Reduced spine density correlates well with impaired LTP (long-term potentiation) at Schaffer collateral-CA1 synapses in acute slices from PLPPR4 KO mice and a lower performance of PLPPR4 KO mice in the Morris-water maze test examining spatial learning (Liu et al., 2016). Mechanistically, the reduced spine density observed in PLPPR4 KO depends on PP2A-mediated activation of integrin (ITGB1) and relates to changes in cellular adhesion (Liu et al., 2016), but not on LPA-dependent LPAR2 signaling (Vogt et al., 2016). For example, the PLPPR4 R346T variant, that is defective in LPA transport, can fully rescue the reduced spine density phenotype of PLPPR4 KO neurons (Liu et al., 2016). Synaptic plasticity is often associated with changes in levels of neurotrophins, particularly BDNF (Leal et al., 2015). Analysis of NGF, BDNF and NT-3 levels in hippocampal tissue of wild type and PLPPR4 KO mice show increased protein levels of NT-3 while NGF and BDNF were not affected (Petzold et al., 2016). The effect of PLPPR4 loss on NT3 levels depends on LPA/LPAR2-signaling (Petzold et al., 2016), suggesting that increased NT3 levels is due to the LPA-dependent hyperexcitability at the glutamatergic synapse, and not associated with the reduced spine density and deficits in LTP.

## Concluding remarks and perspectives

Phospholipid phosphatase-related proteins represent a pleiotropic family of neuronal transmembrane proteins. In this review, we have attempted to organize and categorize their mechanisms of function toward induction of filopodial-type membrane protrusions, their relation to LPA signaling and the implications for neurite growth and branching, axon guidance and regeneration and synaptic transmission and plasticity. Below, we briefly outline four areas of research that we believe will aid in better understanding PLPPR roles in CNS development and disease.

**(1) What can we learn from structural features of PLPPRs?** Structural features that distinguish PLPPRs from each other likely mediate functions specific to PLPPRs while conserved features likely harbor shared functions (Figures 1C,E). The ICD of all PLPPRs, and therefore likely the shared domains of all ICDs such as the proline motif, represent a major determinant of plasma membrane association, induction of filopodia and protein-protein interactions of PLPPRs. Interestingly, long ICDs include a calcium-regulated calmodulin binding site (Tokumitsu et al., 2010). Thus, it is a possibility that PLPPR4 and PLPPR3 functions in synaptic transmission and guidance are regulated also by calcium/calmodulin signaling.

**(2) What is the molecular logic of the PLPPR interaction with LPA signaling?** Currently, we lack the molecular insight necessary to understand the functional implications of the interaction of PLPPRs with LPA signaling. Indeed, several LPA-dependent phenotypes of PLPPRs are compatible with a mode of action where PLPPRs effectively antagonize LPA-induced LPAR activation. However, other studies have highlighted mechanisms compatible with an LPA receptor (or sensor) function (Figure 4). Although the possibility for direct LPA binding to PLPPRs is intriguing (and likely true given the high sequence similarity to LPA-binding PLPPs), it has not been experimentally tested so far. Concerning LPA transport mechanisms, there is currently no consensus of how LPA is taken up by cells (Salous et al., 2013). Rapid clearance of extracellular LPA by endothelial cells appears not to involve dephosphorylation of LPA or transporters of the organic anion transporter family (Salous et al., 2013); therefore, analyses of PLPPR4 dependent LPA-transport could provide insight. An additional question relates to the fate of internalized LPA. In cell lines, uptake of LPA is invariably followed by metabolism (dephosphorylation) to monoacyl glycerol (MAG) (Salous et al., 2013; Vogt et al., 2016) and minor amounts of diacyl glycerol and phosphatidic acid (van der Bend et al., 1992) suggesting that LPA may feed into other lipid-biosynthetic pathways. Future studies aiming at molecular characterization of the LPA-PLPPR interaction will reveal whether they share common modalities and will likely significantly advance the field of LPA pharmacology particularly in the CNS.

**(3) Phospholipid-related protein-induced filopodia: shared or distinct mechanisms?** The filopodia inducing activity of PLPPRs is a unifying feature and has provided a solid framework for investigating their roles in neuron developmental growth and differentiation. It is likely that this cellular activity relates to some extent to PLPPR-dependent regulation of axon guidance, regeneration and synaptic plasticity. The question remains, however, whether PLPPRs engage any of the known molecular machineries of filopodia formation or even whether individual PLPPRs utilize the same mechanisms (Figure 3). There is evidence connecting three PLPPRs (PLPPR1, PLPPR3, and PLPPR5) to phosphoinositide signaling, which is instrumental for activation or recruitment of several filopodia-associated F-actin remodeling proteins (Schink et al., 2016). It is therefore conceivable that PLPPRs could accumulate phosphoinositides and thereby induce several parallel filopodia-forming pathways leading to robustness in filopodia formation.

From a completely different point-of-view, it is intriguing that there is only a handful of transmembrane proteins that have been similarly shown to induce filopodial-type membrane protrusions (Maschietto et al., 2013; Caltagarone et al., 2015; Formoso et al., 2015; Snyder et al., 2015; Ma et al., 2017). In contrast to PLPPRs, these seem to induce filopodia via the known molecular machineries. Of note, certain transmembrane proteins can be selectively concentrated to membrane regions of high positive curvature (as is the case for filopodial membranes), suggesting that they act as membrane curvature sensors (Aimon et al., 2014; Kluge et al., 2022). Alternatively, transmembrane proteins may themselves induce curvature on membranes due to their inherent curvature (e.g., a concave shape) or acquired asymmetry on the membrane (e.g., oligomerization or high local concentration) (McMahon and Gallop, 2005). Theoretically, such initial PLPPR-driven membrane-deforming events could nucleate the actin cytoskeleton. Subsequent recruitment of myosin X, ERM proteins, phosphoinositides, fascin, integrin adhesome components, small GTPases and other filopodia regulators may then further support and strengthen these protrusions. PLPPR-overexpressing cells occasionally exhibit elaborate branched networks of apparently stable – cytoskeleton devoid – membrane protrusions (Yu et al., 2015; Brosig et al., 2019; Tilve et al., 2020). It is interesting to speculate if this is an epiphenomenon of PLPPR-induced physicomechanical alterations in membrane tension and adhesion (Pontes et al., 2017). Further research in some of the above exciting and novel hypotheses will expand our understanding of this PLPPR function and enrich our knowledge on models of filopodia formation.

**(4) Phospholipid phosphatase-related protein relation to human diseases?** Intriguingly, ongoing studies of the phenotypes of PLPPR KO mice and PLPPR polymorphisms in humans clearly suggest the dominant role of PLPPR deficiency for several CNS diseases. This is not restricted only to PLPPR4, for which the phenotypes of KO mice have been by now thoroughly established and include epilepsy, resilience to stress, psychiatric diseases and the regulation of food intake (Trimbuch et al., 2009; Vogt et al., 2016; Schneider et al., 2018; Endle et al., 2022). Further studies on PLPPR3 (Cheng et al., 2016; Brosig et al., 2019), PLPPR1 (Iweka, 2018) and PLPPR5 (Wang et al., 2021) mouse models will likely not only advance our understanding of PLPPR involvement in synaptic transmission and plasticity, circuit assembly, information processing and neuron regeneration, but may extend to effects of LPA signaling. Specifically for psychiatric diseases, anxiety and stress-related disorders, studies have preferably focused on the LPAR1 receptor (Yung et al., 2015). Indeed, LPAR1-deficient mice display a variety of behavioral phenotypes including anhedonia, hypersensitivity to stress, increased anxiety, depression, learning and memory deficiencies as well as pre-pulse inhibition deficits (Yung et al., 2015; Moreno-Fernández et al., 2017). It is thus plausible that some of these LPA-related phenotypes are regulated by PLPPRs during development or even in the adult (Thalman et al., 2018; Birgbauer, 2021). It is also likely that additional pathologies may associate with PLPPR deficiency. A recent histopathological analysis of myopathy in a KO mice cohort suggested the association of PLPPR2 deficiency with muscle lesions characterized by diffuse internalization of myocyte nuclei, and myocyte necrosis (Vogel et al., 2021). In addition, a S12A PLPPR1 polymorphism predicted to destabilize the protein was recently found to be associated with early-onset Parkinson’s disease (Wallen et al., 2018). Finally, several PLPPRs have now been connected to cancer progression and metastasis. Elucidating the roles of PLPPRs in these disease conditions has potential to refine the functions of this protein family. Moreover, being membrane proteins, PLPPRs could also emerge as novel pharmacological targets in disease contexts.

## Author contributions

JF, SB, CK, AP, BJE, and GL contributed equally to this work including writing—review and editing. JF prepared figures. All authors approved the submitted version.

## Footnotes

1
http://phylomedb.org/phylome_104?id=3&seqid=Phy000A3GO&phyid=104&method=VT
